# Is total femoral replacement for non-oncologic and oncologic indications a safe procedure in limb preservation surgery? A single center experience of 22 cases

**DOI:** 10.1186/s40001-018-0302-4

**Published:** 2018-01-16

**Authors:** Andreas Toepfer, Norbert Harrasser, Isabel Petzschner, Florian Pohlig, Ulrich Lenze, Ludger Gerdesmeyer, Ruediger von Eisenhart-Rothe, Heinrich Mühlhofer, Christian Suren

**Affiliations:** 1 Department of Orthopedics and Sports Orthopedics, Technical University of Munich, 81547 Munich, Germany; 2 Sportmedizin Zürich, Schulthess Klinik, Zurich, Switzerland; 3 Departmant of Orthopaedic Surgery and Traumatology, University of Schleswig–Holstein, Kiel, Germany

**Keywords:** Total femoral replacement, Revision arthroplasty, Infection, Non-oncologic megaprosthesis

## Abstract

**Background:**

Several surgical options for the reconstruction of massive bone defects have been described and include biologic methods with autografts and allografts, and the use of tumor endoprostheses (total femoral replacement, TFR). Several types of modular TFR are available, but nevertheless unpredictable outcomes and high complication rates have been described from most authors. The present study aims to compare results after TFR performed with modular total femur prosthesis MML (Fa. ESKA/Orthodynamics) in patients with and without malignant disease.

**Methods:**

Retrospective chart review and functional investigation (Musculoskeletal Tumor Society (MSTS) score, Harris Hip Score (HHS), Oxford Knee Score (OKS), SF-12 Health Survey, and failure classification according to *Henderson*) of TFR cases from 1995 to 2011. Indications for TFR were malignant tumor resection from the femur (*n* = 9, Group A) or failure of a revision arthroplasty without history of malignant disease (*n* = 13, Group B).

**Results:**

Thirty-six patients were treated during the study period, of whom 22 could be investigated clinically after a mean follow-up of 63 months. Overall failure rate for TFR was 59.1%, leading to 38 surgical revisions. The most common failure mechanisms were Type I (soft tissue), followed by Type IV (infection) and Type III (mechanical failure). Mean MSTS score out of 30 was 13 (range 1–25), with significantly higher scores in Group A (mean 19, range 3–25) than Group B (mean 9, range 1–15).

**Conclusion:**

TFR is an established procedure to restore femoral integrity. However, complication rates are considerably high, and depend mainly on the age at initial reconstruction.

## Background

Several surgical techniques for the reconstruction of extensive bone defects have been described and include biologic options, and the use of tumor endoprostheses [1]. Such endoprosthetic bone and joint replacements have developed from customized devices to modern implants with a variety of modular options to replace massive bone defects [2]. At the level of the femur the replacement of the whole bone including the hip and knee joint is an extreme example for limb preservation surgery in modern tumor prosthetics. Total femoral replacement (TFR) is able to reconstruct femoral integrity and usually patients resume mobilization. It comes as little surprise that functional capacity of TFR is compromised compared to conventional hip or knee prostheses, but nevertheless its function is deemed superior to hip disarticulation. Even though several authors reported their results of TFR [1, 3–16], so far many questions are still on debate: It is still unclear, which patients are at risk to experience low functional outcome after this procedure. Hence, it is not well understood, whether complications after TFR depend on the indication for the surgery (e.g., failure of revision arthroplasty or tumor disease) or the age of the patients. Additionally, there have been few reports that compare functional outcomes after TFR for various indications [3, 5, 7, 17, 18].

Therefore, the aim of the present study was to report the results in patients treated with modular total femur prosthesis MML from one orthopedic center. We asked the following questions: (1) Which patients experience a complication or a failure of TFR? (2) Do the complications vary with the indication for TFR (malignant disease vs. revision arthroplasty)? (3) What are the functional outcomes of TFR?

## Methods

**Fig. 1:**
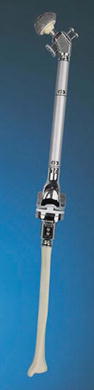
Total femoral prosthesis investigated in the present study (MML, ESKA/Orthodynamics)

**Fig. 2:**
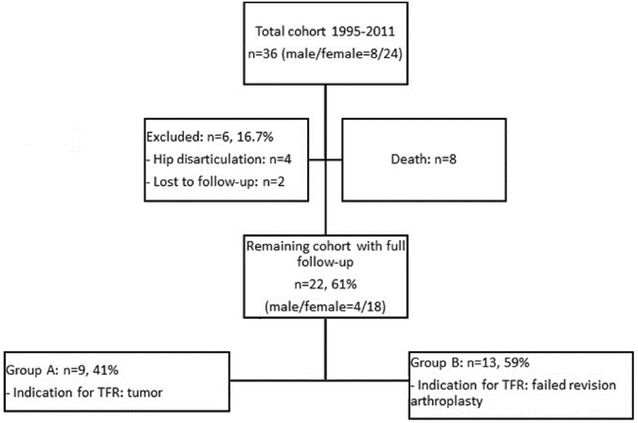
Total cohort and patients included in the study groups

Approval of the respective institutional review boards was obtained before commencement of the study. We retrospectively reviewed our institution’s database for patients with resection of the femur owing to malignant bone tumors or failed revision arthroplasties and defect restauration by TFR from January 1995 to January 2011. Reconstruction of bone defects was performed with a modular total femur prosthesis (MML, ESKA/Orthodynamics, Luebeck, Germany; Fig. 1) comprising a monopolar femoral head component and a fully constrained total knee system. Our database research revealed 36 patients (36 implants) with TFR. Twelve were excluded (eight died from malignant disease, four sustained a hip disarticulation due to persistent periprosthetic infection). Of the 24 remaining patients, two were lost to follow-up within 6 months of surgery. Thus, a total of 22 patients were included in our study (Fig. 2). These patients were contacted by telephone, interviewed, and clinically assessed. Demographic data of the cohort are given in Table 1. Patients were subdivided into Groups A and B according to the indication for TFR: malignant musculoskeletal disease (Group A; *n* = 9; mean age 47 (36–82) years) or failed revision arthroplasty (Group B; *n* = 13; mean age 73 (64–90) years). Surgical details, follow-up, complications, and functional scores for massive bone defect reconstruction [Musculoskeletal Tumor Society (MSTS) score] were recorded. Additionally, functional scores evaluating results after hip and knee surgeries [Harris Hip Score (HHS), Oxford Knee Score (OKS)], pain [visual analogue scale (VAS)], and overall health-related-quality of life (SF-12 Health Survey) were analyzed. At the latest follow-up of patients of Group A, eight (89%) were continuously disease free, and one (11%) was alive with disease (multiple metastases). Complications were analyzed according to the classification proposed by Henderson et al. [19]: Type I is soft tissue failure (e.g., instability of the prosthesis, tendon rupture or avulsion, aseptic wound dehiscence); Type II is aseptic loosening with clinical and radiographic signs of loosening; Type III is structural failure, including periprosthetic fracture or device failure or deficient osseous supporting structure; Type IV is periprosthetic infection requiring removal and subsequent reimplantation of the implant; Type V is tumor progression.

**Table 1:** TFR patient demographics

Demographic	All patients (average ± standard deviation)	Group A: oncologic patients	Group B: failed arthroplasty patients
Age at reconstruction (years)	66 ± 20 years (range 36–90)	47 ± 18 years (range 36–82)	73 ± 8 (range 64–90)
Sex (male/female)	4/18	3/6	1/12
Height (cm)	164 ± 9	163 ± 6	165 ± 7
Weight (kg)	77 ± 10	75 ± 5	78 ± 11
BMI (kg/m^2^)	28.8 ± 4.4	28 ± 4	29.1 ± 6.1
Mean follow-up (months)	63 ± 37 months (range 13–152)	59 ± 48 months (range 13–152)	62 ± 30 months (range 22–110)
Side of TFR (right/left)	12/10	6/3	6/7

## Statistics

Survivorship analysis was performed using the Kaplan–Meier survivorship method. All data are reported as the mean, range, and percentage, where applicable. Comparisons of patient-reported outcomes were performed using a *t* test for unpaired samples. Statistical significance was set at *p* < 0.05. Correlations between numerical data were done with linear regression analysis, and Pearson’s correlation coefficient (*r*) is reported. Statistical analysis was performed using SPSS 2.0 (IBM, Armonk, NY, USA).

## Results

### Indication for TFR

All patients in either group had reported of one or more surgical procedures (nail, primary hip and/or knee prosthesis or megaprosthesis) before implantation of TFR. Various indications for prosthetic reconstruction with TFR are given in Table 2. In Group A, 14 revision surgeries were observed in a total of four individuals, which corresponds to 1.4 (median = 0; range 0–7) revisions per patient prior to TFR. All other patients were converted to TFR from subtotal (proximal (PFR) or distal (DFR) femoral replacement) during the first revision surgery. In Group B, five patients had received a modular megaprosthesis after multiple failed revision procedures in the past. Only two patients received their TFR as a result of the first revision surgery. Hence, 11 patients had a history of 33 revision surgeries prior to their TFR, resulting in 2.4 (median = 2; range 0–8) revisions per patient. The difference between both groups was not significant (*p* = 0.18).

**Table 2:** Medical history and functional outcome according to MSTS score

Patient number	Group/diagnosis		Initial reconstruction	Indication for TFR	Number of revisions prior to TFR	Age at reconstruction	MSTS score						
							General criteria			Limb-specific criteria			Score
							Pain	Function	General acceptance	Supports	Walking ability	Gait	
1	Group A: oncologic patients	OSa	DFR	Mechanical failure	0	62	4	3	4	3	5	4	23
2		OSa	PFR	Periprosthetic fracture	1	31	5	3	1	5	5	3	22
3		OSa	DFR	Mechanical failure	7	44	4	3	3	4	3	3	20
4		OSa	DFR	Mechanical failure	2	34	5	4	4	4	5	3	25
5		OSa	DFR	Aseptic loosening	0	40	3	3	2	5	4	3	20
6		OSa	DFR	Mechanical failure	4	33	3	2	5	5	5	2	22
7		CSa	DFR	Aseptic loosening	0	33	4	3	5	5	4	2	23
8		CSa	HP	Recurrence tumor	0	76	4	2	3	0	3	2	14
9		M	Nail	Metastasis	0	71	0	1	1	0	0	1	3
10	Group B: patients with failed arthroplasty		HP	Periprosthetic fracture	2	64	4	3	3	0	3	1	14
11			HP and KP	Periprosthetic fracture	7	70	2	2	4	0	3	2	13
12			KP	Aseptic loosening	1	84	0	1	0	0	2	1	4
13			KP	Periprosthetic fracture	8	86	1	0	0	0	0	0	1
14			DFR	Periprosthetic fracture	0	74	1	1	0	2	3	1	8
15			Nail	Periprosthetic fracture	4	90	4	0	1	0	1	0	6
16			HP and KP	Periprosthetic fracture	1	79	1	1	3	0	2	1	8
17			KP	Periprosthetic fracture	2	70	3	2	3	0	4	3	15
18			DFR	Periprosthetic fracture	2	85	1	1	0	0	2	1	5
19			PFR	Periprosthetic fracture	0	88	4	0	3	0	0	0	7
20			HP	Septic loosening	1	70	2	1	3	0	2	1	9
21			HP	Periprosthetic fracture	2	83	2	2	4	1	3	3	15
22			HP and KP	Periprosthetic fracture	3	77	4	1	2	0	2	1	10
All patients			Mean		2.1	65.6	2.8	1.8	2.5	1.5	2.8	1.7	13
			Standard deviation		2.5	20.2	1.6	1.2	1.6	2.1	1.6	1.2	7
Group A			Mean		1.6	47.1	3.5	2.6	3.0	3.5	3.6	2.4	18.6
			Standard deviation		2.5	17.7	1.6	0.9	1.6	2.2	1.7	0.7	7.1
Group B			Mean		2.5	78.5	2.1	1.0	1.9	0.3	2.0	1.2	8.4
			Standard deviation		2.5	8.3	1.4	0.7	1.6	0.6	1.2	1.0	4.3

*MSTS* Musculoskeletal Tumor Society, *OSa* osteosarcoma, *CSa* chondrosarcoma, *M* metastasis, *HP* hip prosthesis, *KP* knee prosthesis, *DFR* distal femoral replacement, *PFR* proximal femoral replacement, *TFR* total femoral replacement

### Complications

The mean time from operation to the development of a complication (according to *Henderson*) was 13.4 (0–119) months, with a mean time to complication of 24 (0–119) months for Group A and 6 (1–9) months for Group B. Time to complication varied according to failure mode: Type I presented at an average of 12 (0–54) months after surgery, Type III at 44 (40–48) months, and Type IV at 50 (4–119) months. Type II and V failure were not observed in any of the patients. Overall, there were 20 implant-related complications in 14 patients (64%) with all of these being Type I, III or IV failures (Table 3). Complications yielded to 38 revision surgeries and an overall failure rate for TFR of 59.1%.

**Table 3:** Number of implant failures in the present series as classified according to Henderson et al. [13]

Type of failure	Group A (*n* = 9)	Group B (*n* = 13)	Total number of complications
I (soft tissue failure)	3/9 dislocations 2/9 wound healing problems 1/9 arthrofibrosis	2/13 dislocations 4/13 wound healing problems 1/13 arthrofibrosis	5 6 2
II (aseptic loosening)	–	–	–
III (structural)	1/9 breakage of bolt	1/13 breakage of bolt	2
IV (infection)	1/9	4/13	5
V (tumor progression)	–	–	–
Total	8	12	20

20 complications were found in 14 patients (some patients had multiple failures)

### Analysis of complication types

Type I (soft tissue failure): recurrent hip dislocations were reported in five patients (two in Group A, three in Group B), all of whom were reduced without surgery and underwent conservative treatment. Two patients were treated surgically with three subsequent revision procedures (head replacement, inlay and head replacement, and cup replacement). Wound healing problems were reported in six patients (two in Group A, four in Group B) ending up in ten surgical interventions in five patients. Knee arthrofibrosis was present in one case in each group.

Type III (structural failure): a mechanical failure of the TFR was observed in one patient of either group. In both patients, a failure at the level of the knee system was observed. Both knee modules had to be replaced.

Type IV (deep infection): septic complications of the TFR were observed in five patients (one in Group A, four in Group B) with 21 revision interventions.

### Implant survival analysis

**Fig. 3:**
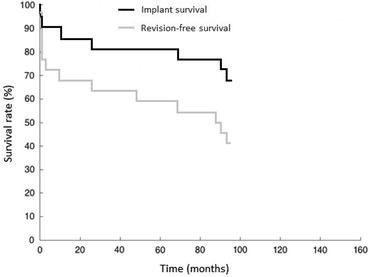
Kaplan–Meier survival analysis. 5-year TFR implant survival = 81.8%; 5-year revision-free survival = 59%

Twenty-two patients were included in the survivorship analysis using Kaplan–Meier curves (Fig. 2). Implant failure (i.e., exchange of prosthetic modules due to implant-related complications) was detected in 18.2% (four cases of 22) of all TFR at an average follow-up of 5 years after primary reconstruction. Another three implant failures occurred after the fifth year from implantation, raising the failure rate to 7/22 (31.8%). Complications led to a partial or total exchange of the prosthesis after an average of 14 months from TFR. Uneventful implant survival was observed in only nine (40.9%) of the 22 patients (Fig. 3).

### Clinical outcome

Patients in the present study had a mean VAS value (Maximum: 10, Minimum: 0) of 5.7 preoperatively (Group A: 4.5; Group B: 5.8) and 3.4 after TFR (Group A: 1.9, Group B: 4). Difference was statistically significant between pre- and postoperative VAS values within each group (*p* = 0.04), and between groups for pre- and postoperative values (*p* = 0.01).

The mean MSTS score (out of 30) across both groups was 13 (43%, range 1–25). Scores in Group A (19 (64%), range 3–25) were significantly (*p* = 0.003) higher than in Group B (9 (30%), range 1–15). Sub-score analyses revealed significant differences between the groups in function (*p* = 0.002), supports (*p* = 0.001), walking ability (*p* = 0.007), and gait (*p* = 0.005). Statistical analysis revealed a strong negative correlation between age and clinical outcome (MSTS) after surgery (*r* = − 0.86). On the other hand, only a weak negative correlation was found for number of revision surgeries prior TFR and clinical outcome (MSTS) after surgery (*r* = − 0.11).

Clinical outcome data computed by HHS and OKS, as well as results of SF-12 analysis are given in Table 4.

**Table 4:** Functional outcome results of both groups

Items	Group A [value, (range)]	Group B [value, (range)]	*p* value
HHS			
Mean score	69.7 (12–88)	35.4 (15–57)	0.007
Pain	31.6 (0–44)	17.7 (10–40)	0.002
Function	21 (0–30)	6.2 (0–14)	0.001
Activity	10 (5–12)	5.2 (0–10)	0.002
Contractures	3.9 (3–4)	3.7 (3–4)	0.6
Motion	3.2 (2–5)	2.7 (2–4)	0.2
OKS			
Mean score	26.2 (5–39)	15.3 (4–26)	0.03
SF-12			
Physical subdomain	38.3 (21.9–50.1)	28.1 (21.3–35.6)	0.02
Mental subdomain	52.5 (10.5–62.7)	48.5 (27.3–62.9)	0.2

*p* < 0.05 = significant; HHS (Harris hip score): < 70: poor; 70–79: fair; 80–89: good; 90–100: excellent OKS (Oxford knee score): < 19: poor; 20–29: fair; 30–39: good; 40–48: very good SF-12 (Short Form 12 Health Survey): healthy controls > 50

## Discussion

Reconstruction of massive bone defects of the femur after oncologic resection or failed revision arthroplasty represents a challenge for orthopedic surgeons. Here, we analyzed 36 TFRs, and reviewed the clinical and functional outcomes of 22, in patients with a history of malignant diseases (Group A) or failed revision arthroplasties (Group B). To the best of our knowledge, there is no published case series using a standardized failure-mode classification of TFRs performed with the MML system (Fa. ESKA/Orthodynamics).

One of the main findings of the present study is the significant difference in clinical outcome between the groups (Table 2). Linear regression analysis with the potentially most influential factors for clinical outcome (age, surgeries prior TFR) showed a strong negative correlation only for age. This finding partially agrees with previous studies showing significant differences between indication for TFR, age at TFR and number of revisions prior to TFR [19, 20]. The oncological cohort in the present study showed high rates of disease-free survival. Six patients with osteosarcoma (mean follow-up: 7.7 years) and two patients with chondrosarcoma (mean follow-up: 1.8 years) were continuously disease free. Comparing the drop-outs (*n* = 8; all patients died of progressive tumor disease) of the initial cohort of 36 patients and patients of Group A (eight still alive without recurrence, one with metastatic disease) we found that the local extent of the primary tumor and the presence of metastases were negatively correlated with survival. In all drop-outs a local extend of the tumor of more than half the length of the femur was present, while in all patients of group A the initial tumor was less than half of the length of the femur. All patients with initial metastatic disease and primary reconstruction with TFR had died at a mean of 9 month after surgery. Of the eight drop-outs, one died of an osteosarcoma (G2), one of a chondrosarcoma (G3), one of a pleomorphic sarcoma (G3) and five of metastases due to lung/renal cell/rectum carcinoma. All patients of group A except for one (patient 9: renal cell carcinoma) had suffered from a sarcoma and all were graded three. Hence, grading of the sarcomas did not influence survival in our series. Therefore, it is difficult to provide universal surgical guidelines for decision-making between reconstruction with TFR or hip disarticulation in these patients. It seems that the survival of patients with extensive tumorous disease cannot be improved with either surgical method.

We found an overall non-oncologic complication rate of 59.1% in our patients who underwent TFR (Fig. 3). It should be noted that none of the patients included in the present study (*n* = 22) had a primary defect reconstruction with TFR. In fact, 47 revision surgeries were performed in the study population prior to TFR. Only seven out of 22 patients received their TFR within the first revision of their megaprosthesis or intramedullary nail. Statistical analysis revealed only a weak negative correlation for number of revision surgeries prior TFR and clinical outcome after surgery. This is important because outcome after TFR has not been correlated with prior revision surgeries so far.

Analysis of failures after TFR revealed some differences between the groups. Type I failure (soft tissue failure) was detected in 13 cases necessitating 15 surgical revisions. Wound healing problems (six patients, 27%) and hip dislocations (five patients, 23%) were the most common complications among Type I failures. Incidences of these complications are reported to vary between 0 and 45% [3, 6, 8, 9, 11]. From large series with primary hip arthroplasty, it is known that 75% of dislocations occur within the first 2 months of implantation [21]. In our study, this was observed in 67% of dislocations. To prevent hip dislocation in cases of residual trochanteric bone or viable tendinous abductor structures, we preferred direct attachment to the endoprosthetic implant using non-resorbable sutures. Alternatively, the ligament augmentation reconstruction system (LARS^®^) may be a helpful tool for more stable soft tissue repair in cases of extensive loss [12]. Additionally, promising results regarding hip stability can be obtained if tripolar cups are used [2, 3, 22].

In our series, no Type II failure (aseptic loosening) was found, confirming previous reports of a low incidence of this failure type in TFR [6, 9]. In cases using megaprostheses, this type of failure was reported at a rate of 2.4–15.4% for cemented [23–26] and 0–8% for cementless implants [10, 27–29]. Unlike PFR or DFR, TFR implantation does not rely on diaphyseal stem fixation but uses the common techniques of total hip arthroplasty and (fully constrained) total knee arthroplasty with a standard acetabular cup and tibial metaphyseal stem fixation. This and the lower activity level of patients from the present study might explain the results for aseptic loosening with TFR compared with PFR and DFR.

Structural failure (Type III) was observed in two patients in our series, namely a prosthetic breakage at the level of the hinged knee joint. In both cases, the affected prosthetic parts were replaced and no further material failure was observed. In the literature, the incidence of prosthetic component breakage in megaprostheses is 0–7.7%, with lower incidences in TFR than in PFR/DFR. Again, this might be attributed to the absence of diaphyseal stems in TFR, which are known weak spots in modular megaprostheses [30]. Other authors conclude that the lower mobility and activity in this population is a reason for lower rates of structural failure [10, 23, 25, 26, 28, 29]. In our experience, bolt breakage at the level of the hinged knee module occurred mostly in the first-generation design of the MML prosthesis in patients with DFR, where the whole load is carried by the central axis bolt. This bolt was strengthened in second-generation prostheses, which have been used since the late 1990s [30].

Type IV failure (deep infection) was observed in five patients (22%) in our series. Note that another four cases with hip disarticulation due to persistent periprosthetic TFR infection were excluded from the initial cohort of 36 patients. Hence, a total infection rate of 25% (nine out of 36) was observed in our study. These data are comparable to findings described in the recent literature (0–20% in TFR) [3, 6, 8, 9, 11, 22]. Permanent eradication of infection was achieved in all cases in our cohort with a total of 21 revisions.

Type V (implant-independent) failure was never observed in our cohort. Other authors described rates between 5 and 20% [3, 8, 9, 11].

Analyzing the occurrence of implant-related complications in the course of time, we found some specific differences between the various failure modes according to Henderson: Type I failures (soft tissue) occurred after an average of 12 months after surgery, and included mainly instability or aseptic wound dehiscence. On the other hand, Type III (structural failure) and IV (periprosthetic infection) failures occurred after an average of 44 and 50 months, respectively. The differences between these short-term and mid-/long-term complications is not surprising, taking into account that Type I complications are more related to the surgical procedure itself and Type III complications to the implant. However, these differences have so far not been described in the context of TFR. The finding that Type IV complications occurred rather late in the present study is only partially supported by data in the literature, where early and late occurrence of periprosthetic infections of TFR have been described [5, 7, 14, 22].

Functional outcome measurement with respect to the MSTS score, as the only established score for evaluation of massive bone reconstructions, revealed an average value of 13 (43%) in the present study, inferior to other studies, which reported scores of 17–24 (59–80%) (Table 5). However, consideration of individual cases from both groups is necessary for adequate interpretation: one patient in Group A was in a palliative condition and unable to sit or stand owing to his disease at latest follow-up and two patients in Group B suffered from advanced dementia. Additionally, there was a significant difference of 26 years in the mean ages of the two groups. This is the most influencing factor for MSTS score differences between the groups as shown in linear regression analysis. HHS is a well-established hip score and has so far only been used by Berend et al. to evaluate hip function in TFR [4]. In their series, an average value of 70 was detected. In the present study, an overall score of 49 was identified, also with a significant difference between the groups (Table 4). Evaluation of TFR cannot be compared with results from primary or “conventional” revision total hip arthroplasty. OKS has so far not been used to evaluate TFR. In the present study, a rather low average value (Table 4) was observed, but again there was a significant difference between the groups. As with HHS, OKS does not seem to be an appropriate tool with which to evaluate functional outcome after TFR.

**Table 5:** Comparison of the present results with those from other studies involving TFR indication for TFR: tumor disease, failed arthroplasty or both (= mixed)

Authors	Number of patients	Follow-up (months)	Average age (years)	Indication for TFR	MSTS score (%)	Revision-rate (%)	Survivorship of TFR	Complications requiring surgery (no. patients)
Puri [11]	5/8	33	32	Tumor	80	12.5	88%	Infection (1)
Ruggieri [16]	21/23	48	21	Tumor	66	23.8	One failure	Infection (2), mechanical failure (3)
Kalra [8]	11/26	57	40	Tumor	72	11.5	80% prosthetic survival at 10 years	Loosening (1), dislocation (3), deep infection (2), foot drop (1), amputation (2)
Natarajan [9]	17/17	54	31	Tumor	67	23.5	82.4%	Infections (2), Hip dislocations (2)
Ahmed [3]	4/9	51	47	Tumor	72	33	66%	Infection (2), tibial component lossening (1)
Amanatullah [14]	20	73	65	Failed arthroplasty	NA	30	70% at 5 years	Infection (7), hip dislocation (5), limb length discrepancy (2), knee flexion contracture (1)
Berend [4]	58/59	58	74	Failed arthroplasty	NA	30.5	65% at 5 years	Infection (8), hip dislocation (7), tibial component loosening (2), acetabular component loosening (1)
Fontain [6]	12/14	90	63	Failed arthroplasty	59	35.7	NA	Hip dislocation (5), infection (3)
Friesecke [7]	81/100	59	68	Failed arthroplasty	77	21	NA	Infection (12), hip dislocation (6), prosthesis failure (3), patellar issues (2), hematoma (2), peroneal nerve palsy (1), delayed wound healing (1)
Lombardi [1]	50/75	42	73	Failed arthroplasty	NA	30.7	NA	Infection (11), hip dislocation (7), tibial component loosening (2), acetabular component loosening (1), hematoma (1), periprosthetic fracture (1)
Mankin [15]	15	54	52	Mixed	NA	33.3	NA	Prosthesis failure (4), infection (1)
Nerubay [17]	7/19	18–96	20	Tumor	NA	NA	NA	Wound healing problems (10), infection (1), popliteal vein injury (1), prosthesis failure (1)
Steinbrink [18]	32	6–84	56	Mixed	NA	9.4	NA	Infection (2), hip dislocation (1), prosthesis failure (1), patellar pain (1)
Ward [16]	11/21	31	44.6	Mixed	NA	2.4	NA	Infection (3), hip dislocation (2), patellar pain (1)
Current study	22/36	63	66	Mixed	43	59.1	81.8% at 5 years	Infection (5), hip dislocation (2), wound healing problems (5), prosthesis failure (2), arthrofibrosis (2)

Number of patients (*x*/*y*): number of patients included in study/total number of patients including drop-outs

In summary, VAS and functional outcome measures revealed a significant reduction in pain after TFR compared with preoperative values. Function with mobility was reduced in both groups, but significantly better results were observed in patients from Group A. This finding was supported by the physical SF-12 survey score. The differences in the mental SF-12 survey scores were not significant, stating good acceptance of the TFR in both groups. Patients in the present study communicated clearly that regaining partial mobility and reduction of pain are the most important items for achieving satisfaction after TFR. This finding has already been published by other authors [4, 6].

This study has several limitations. First, the retrospective design is subject to recall and selection bias. The number of patients is quiet small and statistical analysis is, therefore, difficult. However, owing to the rare indication for this procedure, our series is comparable to studies published so far. Second, the study lacks a true control group, meaning we cannot directly compare our results with other types of implants or biologic reconstructions. Third, the differences between groups regarding age, prior revision surgeries and varying diagnosis make comparison difficult.

## Conclusion

This retrospective analysis of our series confirmed the high incidence of implant-related complications and failures in TFR for complex oncological and non-oncological lower limb salvage as already outlined by previous studies with different endoprosthetic systems. Infection and soft tissue failure were the most frequent modes of failure. Implant survival of 81.8% at 5 years was observed. However, only 40.9% of all TFRs had an uneventful survival at latest follow-up. Clinical outcome seems to depend mainly on the patients’ age at reconstruction of bone defect with TFR. Our data suggest that indication for TFR remains a salvage procedure for limb preservation. Therefore, this procedure should only be considered when the alternative is hip disarticulation and the patient should be aware of the potential high complication rates of this massive reconstruction.
